# Pragmatic Competence and Willingness to Communicate Among L2 Learners of Chinese

**DOI:** 10.3389/fpsyg.2021.797419

**Published:** 2021-12-06

**Authors:** Xiaoxuan Lv, Wei Ren, Lin Li

**Affiliations:** ^1^School of Foreign Languages, Beihang University, Beijing, China; ^2^School of International Studies, University of International Business and Economics, Beijing, China

**Keywords:** pragmatic awareness, pragmatic comprehension, willingness to communicate, self-perceived communication competence, Chinese as second language

## Abstract

Research in second language (L2) pragmatics has paid increasing attention to learners’ individual differences, but few studies have examined the relationship between learners’ willingness to communicate (WTC) in L2 and their pragmatic competence. To this end, this study investigates the association between WTC and pragmatic awareness and comprehension of Chinese as a second language (CSL) learners. A total of 80 CSL learners studying abroad in three universities in China participated in this study. Data were collected through a WTC questionnaire, a self-perceived communication competence (SPCC) questionnaire, a pragmatic awareness judgment task, and a multiple-choice test for pragmatic comprehension. Statistical analyses were conducted to explore the relationship between the learners’ pragmatic awareness and pragmatic comprehension on the one hand and their WTC and SPCC in L2 on the other. The findings indicated that SPCC correlated positively with the learners’ L2 pragmatic comprehension, but not with their L2 pragmatic awareness. No correlation was found between WTC and pragmatic awareness and comprehension. The results suggest that SPCC may contribute to learners’ L2 pragmatic comprehension; some implications for teaching and future research directions are also discussed.

## Introduction

Pragmatic competence is key to effective communication and success in second language (L2) learning. However, developing pragmatic competence can bring challenges for L2 learners, regardless of their language proficiency. Even those with advanced abilities in a L2 sometimes use the language inappropriately in daily communication due to their lack of pragmatic knowledge (Bardovi-Harlig and Dörnyei, 1998; Bardovi-Harlig, 2001; Ren, 2013). Studies on L2 pragmatics have started to focus on L2 learners’ differences (Roever et al., 2014; Takahashi, 2019; Yang and Ren, 2019). Willingness to communicate (WTC), which is a learner’s intention to speak in the target language given free choice (MacIntyre, 2020; MacIntyre et al., 2020), is a factor that influences individual differences in language learning (Henry et al., 2021). WTC is found as a vital factor for communication (Cao, 2011; MacIntyre, 2020). Some studies have identified several factors that influenced WTC in L2. For example, Liu (2017) investigated adult Chinese as a second language (CSL) learners’ WTC in Chinese and found their WTC was determined by speaking anxiety and length of stay in China. Nkrumah (2021) also found that the environment and teaching practices influenced CSL learners’ WTC. However, aside from Hosseinpur and Nevisi (2017), which found a positive relation between pragmatic production and WTC, few studies have investigated the relationship between pragmatic competence and WTC. Indeed, little attention has been paid to L2 learners’ WTC from the perspective of pragmatics. In addition, previous studies have found that self-perceived communication competence (SPCC) is one of the most highly correlated factors with WTC (e.g., Yu, 2009). Thus, this study will examine the relationship between pragmatic competence and WTC, including SPCC.

Pragmatic awareness and pragmatic comprehension are important components of receptive pragmatic competence (Ren, 2015). Many previous studies on pragmatic awareness have paid attention to influencing factors such as proficiency and study abroad (e.g., Bardovi-Harlig and Dörnyei, 1998; Schauer, 2009; Ren, 2015), but only a few have focused on L2 learners’ individual differences, such as motivation (e.g., Tagashira et al., 2011; Takahashi, 2015; Yang and Ren, 2019) and social networks (Li et al., 2021). This is partially because learners were considered as a homogeneous group in pragmatic awareness research for a long time (Barron, 2019; Yang and Ren, 2019). There is increasing awareness that L2 learners’ inner characteristics, i.e., individual differences such as learners’ attitudes and identities, vary and influence the development of pragmatic awareness (LoCastro, 2001; Hassall, 2015; Ren, 2018). Previous findings have also shown that even when learners are able to comprehend implied meaning in L2, their performance varies in different types of implicatures (Taguchi et al., 2013). L2 learners’ comprehension of these implicatures is influenced by pragmatic awareness (Alcón Soler and Jordà, 2008). A systematic investigation of the relationship between pragmatic comprehension and other dimensions of individual differences, for example, WTC/SPCC, will therefore contribute to studies on learners’ L2 pragmatic competence development.

Although CSL learners’ pragmatic production has been investigated (e.g., Qi and Lai, 2017; Ren, 2019; Gong et al., 2020b), few studies have examined their pragmatic awareness and comprehension. On the other hand, some studies have explored how CSL learners’ individual differences (motivation, attitude, etc.) influence their language acquisition and use (Ma et al., 2017; Gong et al., 2018b, 2020a), but little attention was paid to their pragmatic competence. Therefore, it is necessary to investigate the impact of individual differences, for example, WTC and SPCC, on L2 learners’ pragmatic awareness and pragmatic comprehension, particularly in a language other than English such as Chinese. Therefore, this study aims to investigate the pragmatic awareness and comprehension of CSL learners and to explore the relationship between their pragmatic awareness/comprehension and WTC/SPCC.

## Literature Review

### Willingness to Communicate and Self-Perceived Communication Competence

The construct of WTC in L2 has been defined as “a readiness to enter into discourse at a particular time with a specific person or persons, using a L2” (MacIntyre et al., 1998, p.547). It is believed that WTC is a complex construct because of its situated nature and context-dependent characteristics (MacIntyre, 2020). Since L2 WTC is situated in nature and it changes when L2 learners interact with their environment (MacIntyre et al., 2011), WTC research pays much attention to contextual influences. Given the importance of contexts in pragmatics, a link between WTC research and pragmatics research could be established (MacIntyre et al., 2020).

Numerous studies on the associations between L2 WTC and other variables at the social or personal level have been explored by quantitative methods and questionnaires (Lahuerta, 2014; Peng, 2014; Zhou et al., 2020). Some individual difference factors such as L2 attitude, SPCC, L2 communicative confidence, and communication anxiety have been investigated and found to have an impact on WTC (Lockley, 2013; Lahuerta, 2014; Zhou et al., 2020). Among these variables, SPCC has been the most frequently examined and has a relatively strong impact on WTC (Burroughs et al., 2003; Lockley, 2013; Shirvan et al., 2019). Thus, SPCC will also be examined in this study.

Self-perceived communication competence reflects learners’ self-assessment of their L2 competence (MacIntyre, 1994; Peng, 2014). Previous studies on L2 WTC have shown that there is a close relationship between WTC and SPCC (Yashima, 2002), in that SPCC appears to be a strong predictor of WTC with others in L2 (Clément et al., 2003; Lockley, 2013; Shirvan et al., 2019). Yu and Hsu (2008) argued that L2 learners with a high level of SPCC would like to communicate more. As a predictor of WTC, SPCC also promotes or hinders communication. Thus, to ensure SPCC positively promotes L2 learning, studies have found that crucial elements such as learning environment, teachers’ attitudes, and approaches should be given serious consideration (Horwitz, 2001; Ushioda, 2010).

The interaction between SPCC and actual L2 competence has also been investigated (Lockley, 2013). L2 learners’ SPCC is found more decisive for WTC than their actual L2 competence (MacIntyre et al., 1998). The learning context and language experiences are found to be associated with the effect of SPCC on L2 WTC (Shirvan et al., 2019). Individual differences such as gender and age have also been examined, which are shown as significant moderators in the relationship between SPCC and actual L2 competence (Lahuerta, 2014). However, most studies on WTC have been conducted with learners of English as a L2, while few studies have focused on learners of Chinese as the target language.

### Pragmatic Awareness and Pragmatic Comprehension

Pragmatic awareness, a crucial dimension of receptive pragmatic competence (Ren, 2015), is defined as “conscious, reflective and explicit knowledge about pragmatics” (Alcón Soler and Jordà, 2008, p.1948). A number of studies have investigated the impact of different variables on the development of pragmatic awareness (Bardovi-Harlig and Dörnyei, 1998; Schauer, 2006), which was found to correlate positively with attitudes towards the L2 community (Yang and Ren, 2019). Length of residence in the target community was also shown to be positively related to the development of pragmatic awareness (Schauer, 2006). Meanwhile, an examination of L2 learners’ pragmatic awareness revealed some linguistic factors that contributed to the conditions needed to understand pragmatic meaning (Alcón Soler and Jordà, 2008). L2 learners’ awareness of pragmatic features may be assessed by meta-pragmatic judgment tasks (Bardovi-Harlig, 2018), which have been demonstrated to be a reliable assessment tool for pragmatic awareness. In this study, we will adopt this technique to assess CSL learners’ pragmatic awareness.

Pragmatic comprehension is key to interpreting mechanisms of interpersonal communication (Taguchi and Yamaguchi, 2019), which refers to the ability to comprehend implied meaning in the target language (Taguchi, 2008). L2 learners show differences in their pragmatic comprehension of different implicature types, since the implicatures incorporate different levels of conventionality. The ability to comprehend implicatures is mediated by L2 proficiency and learning environment in terms of both accuracy and speed of comprehension (Taguchi et al., 2013). Taguchi (2011) examined learners’ pragmatic comprehension and found that study-abroad experience had a greater impact on the comprehension of conventional expressions than non-conventional ones.

Based on previous research (e.g., Taguchi et al., 2013; Li, 2018), in this study we will develop a framework of implicatures with three types, consisting of conventionally indirect refusals, conventional routines, and non-conventionally indirect opinions. For pragmatic comprehension assessment, most studies have adopted a reading test where prompts take the form of a sentence or dialogue and multiple-choice questions, although some studies have used audio input or video clips in a listening test (Taguchi and Yamaguchi, 2019). This study will investigate L2 learners’ comprehension of implicatures based on a reading test.

The above review reveals that few studies have investigated WTC and SPCC in L2 pragmatics and the field of learning and teaching Chinese as a L2. It is an open question whether pragmatic awareness and pragmatic comprehension are related to CSL learners’ WTC and SPCC, and to what extent. The following research questions will therefore be addressed:

(1) To what extent can the CSL learners complete the pragmatic awareness and pragmatic comprehension tasks?

(2) Are there any correlations between pragmatic awareness/pragmatic comprehension and the WTC/SPCC of the CSL learners?

## Methodology

This study employs a quantitative design using a set of questionnaires to explore the relationships between pragmatic awareness/pragmatic comprehension and WTC/SPCC.

### Participants

Since a quantitative examination needs a fair number of participants, the study was conducted in different universities in China to recruit participants with convenience sampling. A total of 90 CSL learners from various countries volunteered to participate in the study. After validating demographic information, it was found that two learners had not yet come to China due to the influence of COVID-19, and eight learners’ first language was Chinese. Thus, their data were excluded from the analysis. Therefore, data from 80 learners were analyzed in this study (45 males and 35 females, *Mean age* = 23.56, *SD* = 4.706). They came from different countries, including Russian, the United Kingdom, the United States, Korea, Thailand, Poland, Mali, Japan, etc., and were majoring in various disciplines such as economics, chemistry, physics, and so on. The learners had learned Chinese for nearly four years on average (*M* = 6.23, *SD* = 5.40). 90% of them had lived in a Chinese-speaking country above one year; 68.7% of them had taken and passed HSK (28 learners at level 6, 19 at level 5, 6 at level 4, 1 at level 3, and 1 at level 2).

### Instruments

Data were collected by a web-based survey consisting of demographic information (gender, name, how many years they had studied Chinese, etc.), a WTC questionnaire, a SPCC questionnaire, a pragmatic awareness judgment task, and a multiple-choice test for pragmatic comprehension.

#### Willingness to Communicate Questionnaire

The WTC questionnaire consists of 12 items adapted from MacIntyre et al. (1998). While the WTC items were originally developed by McCroskey (1992) in the context of L1, MacIntyre et al. (1998) adapted them into a L2 setting. We adapted the items from asking students their WTC in English to their WTC in Chinese. The learners were asked to indicate the percentage of time (from 0 to 100%) they would choose to communicate in Chinese in different situations; some item examples are “Talk with an acquaintance” and “Talk in a small group of strangers” (see Supplementary Appendix I). The internal consistency reliability of the WTC questionnaire was high (α = 0.93).

#### Self-Perceived Communication Competence Questionnaire

The SPCC questionnaire was designed to measure learners’ self-perceived level of communication competence in different contexts, with 12 items adapted from MacIntyre et al. (1998). The learners were asked to indicate the percentage of time (from 0 to 100%) they considered they were able to communicate in Chinese in different situations. Some item examples are “Talk with an acquaintance” and “Talk in a small group of strangers” (see Supplementary Appendix II). The internal consistency reliability of the SPCC questionnaire was high (α = 0.95).

#### Pragmatic Awareness Judgment Task

The pragmatic awareness judgment task, containing ten items, was adapted from Bardovi-Harlig and Dörnyei (1998). Since the task was originally developed for English as the target language, we translated all the items into Chinese to suit our research purpose in CSL. Six native Chinese speakers piloted this instrument, and two items were replaced. Later, another two native Chinese speakers piloted the adjusted instrument and no item was needed to be revised. Each item contained a brief Chinese description of a scenario, a two-turn dialogue, and a question prompt; learners were asked to judge whether the final sentence of the short exchange was appropriate or not. If not, they should further judge the degree of inappropriateness, using scores of 0 for “not at all” to 6 for “extremely seriously.” The test items included two categories: (a) sentences that were pragmatically appropriate, and (b) sentences that were pragmatically inappropriate (see Supplementary Appendix III). The internal consistency reliability of the pragmatic awareness judgment task was acceptable (α = 0.75).

#### Multiple-Choice Test for Pragmatic Comprehension

The multiple-choice test for pragmatic comprehension was adapted from the pragmatic comprehension test used in Alsuhaibani (2020), which was a written version of the pragmatic listening test originally developed by Taguchi (2012). In addition, we also consulted Li (2018) in developing the items. The test, containing 12 items, was composed of conventional implicatures (4 items for routines and 4 items for indirect refusal) and non-conventionally indirect opinions (4 items) (see Supplementary Appendix IV). All the items were piloted by native Chinese speakers and minor changes in expressions were made. The internal consistency reliability of the multiple-choice test for pragmatic comprehension was acceptable (α = 0.80).

### Procedure

Web-based questionnaire data were collected to assess learners’ levels of WTC, SPCC, pragmatic awareness, and pragmatic comprehension. Learners received a link to the web-based questionnaires from their instructors. All learners were recruited voluntarily and assured that their participation would not affect their term grades.

### Data Analysis

The scores from the 80 learners were imported into SPSS version 22.0 for analysis. The WTC and SPCC questionnaires were coded according to the percentage selected by learners, and total points were computed.

Each answer in pragmatic awareness judgment task was scored out of 2 points. If the sentence was appropriate, the participant was given 2 points for selecting ‘appropriate’ and 0 point for ‘inappropriate.’ If the sentence was inappropriate, the item was scored into two parts, 1 point for selecting ‘inappropriate’ and 1 point for accurately judging the degree of appropriateness.

Each answer in the multiple-choice test for pragmatic comprehension was coded “1” for a selection of the desired answer and “0” for other options. This means that a score of 12 was the highest score the learners could achieve. Descriptive analysis was conducted. Correlations between pragmatic awareness/pragmatic comprehension and WTC/SPCC were investigated separately. In order to examine the interactions between variables, a moderate effect analysis was also conducted.

## Results

### Pragmatic Awareness and Pragmatic Comprehension

#### Pragmatic Awareness

As previously stated, there were 10 items in the pragmatic awareness judgment task, with a possible score of 2 marks for each item. Thus, the highest possible score on the task was 20 points. Most learners performed well in this task, with an average score of over 13 points (*M* = 13.04, *SD* = 3.513). The results showed that all the CSL learners were good at identifying pragmatic errors and judging the degree of inappropriateness.

Table 1 presents the means and standard deviations of each item in the pragmatic awareness judgment task. The results showed that Item 8 was the easiest for the learners while Item 6 the most difficult. Most learners could judge the appropriateness of Item 8, and they all consider the utterance “*我觉得已经很好了,非常感谢您提供的所有信息*” (“That’s great. Thank you so much for all the information”) addressed to a teacher to be an appropriate expression. However, they encountered difficulties in judging Item 6, most of them failing to correctly judge whether Anna’s utterance to a teacher “*你好！我是安娜。你要是不介意的话,我想让你帮我填一下这个问卷*” (“Hello. My name is Anna. If you don’t mind, I would like you to fill this in for me”) was appropriate or not.

**TABLE 1:** Learners’ performance in pragmatic awareness judgment task (*n* = 80).

Item	*Mean*	*Standard deviation*
8	1.80	0.604
7	1.70	0.719
10	1.70	0.719
9	1.64	0.661
2	1.41	0.760
5	1.40	0.739
4	1.18	0.725
5	0.91	0.845
1	0.71	0.874
6	0.65	0.781

#### Pragmatic Comprehension

As mentioned above, there were 12 items in the pragmatic comprehension task. If learners chose the desired answer, 1 point was scored. Thus, the highest possible score for this task was 12 points. Again, most of the CSL learners performed well in this task, with an overall average score above 9 points (*M* = 9.55, *SD* = 2.951). The results demonstrated that the CSL learners were generally successful at identifying implied meaning in terms of different implicature types.

The means and standard deviations of the learners’ performance for each item are presented in Table 2. Most of the learners could comprehend the implied meaning of the underlined sentence in the dialogue “他可以出去开饭店了!!” (“He can run a restaurant!”) (Item 4). However, learners had great difficulty interpreting the implicature of the comment “就你那手艺啊！” (“Just your cooking!”) (Item 7).

**TABLE 2:** Learners’ performance in the pragmatic comprehension task (*n* = 80).

Item	*Mean*	*Standard deviation*
4	0.91	0.284
1	0.90	0.302
11	0.89	0.318
9	0.88	0.333
12	0.85	0.359
8	0.84	0.371
2	0.81	0.393
3	0.75	0.436
6	0.74	0.443
5	0.70	0.461
10	0.66	0.476
7	0.63	0.487

As shown in Table 3, the learners’ comprehension of implicatures differed in terms of the different types, with their comprehension of routines the lowest. In general, the learners’ comprehension of non-conventional indirect opinion/comments was the best, a bit higher than that of conventional indirect refusals.

**TABLE 3:** The comprehension of different implicatures.

Item	*Mean*	*Standard deviation*
Non-conventional indirect opinions/comments	3.375	0.107
Conventional indirect refusals	3.275	0.128
Routines	2.900	0.141

### Relationship Between Pragmatic Awareness/Comprehension and WTC/SPCC

As presented in Table 4, the correlation coefficient between the CSL learners’ pragmatic awareness and their SPCC indicated a small or no correlation. Also, it was found that there was no correlation between the learners’ pragmatic awareness and their WTC either.

**TABLE 4:** Correlations between pragmatic awareness and WTC/SPCC.

		WTC	SPCC
Pragmatic awareness	Pearson correlation	0.138	0.199
	Sig. (2-tailed)	0.224	0.077
	N	80	80

As presented in Table 5, the learners’ overall pragmatic comprehension demonstrated no relation with their WTC; however, there was a weak correlation between CSL learners’ comprehension and their SPCC.

**TABLE 5:** Correlations between pragmatic comprehension and WTC/SPCC.

		WTC	SPCC
Pragmatic Comprehension	Pearson Correlation	0.058	0.259*
	Sig. (2-tailed)	0.607	0.021
	N	80	80

*Correlation is significant at the 0.05 level (2-tailed).

As aforementioned, there are three types of implicatures in this test, namely conventionally indirect refusals, conventional routines, and non-conventionally indirect opinions. Thus, three subgroups of pragmatic comprehension were investigated.

The results in Table 6 showed that there was a weak correlation between the performance of conventionally indirect refusals and SPCC (*r* = 0.234), but this was statistically significant. In addition, the learners’ performance in routines also was found to be weakly correlated with SPCC (*r* = 0.316). However, there was a small or no correlation between all the three subgroups of implicature and WTC.

**TABLE 6:** Correlations between subgroups of pragmatic comprehension and WTC/SPCC.

		WTC	SPCC
Conventional indirect refusals	Pearson Correlation	0.089	0.234*
	Sig. (2-tailed)	0.434	0.037
	N	80	80
Routines	Pearson Correlation	0.051	0.316*
	Sig. (2-tailed)	0.650	0.000
	N	80	80
Non-conventional indirect	Pearson Correlation	0.006	0.100
opinions or comments	Sig. (2-tailed)	0.958	0.376
	N	80	80

*Correlation is significant at the 0.05 level (2-tailed).

To further explore how pragmatic comprehension interacts with SPCC, based on previous studies the moderating effects of several variables were examined, such as gender, age, etc. Only age was found to be a moderating variable between SPCC and pragmatic comprehension (see Table 7).

**TABLE 7:** Moderate effect analysis (*n* = 80).

	Model 1					Model 2					Model 3				
	*B*	*SDE*	*T*	*p*	β	*B*	SDE	*t*	*p*	β	*B*	*SDE*	*t*	*p*	β
Constant	7.669	0.290	26.471	0.000**	−	7.669	0.290	26.403	0.000**	−	7.554	0.287	26.311	0.000**	−
Pragmatic comprehension	0.234	0.099	2.364	0.021*	0.259	0.221	0.100	2.208	0.030*	0.245	0.235	0.098	2.399	0.019*	0.260
Age						−0.049	0.063	−0.774	0.441	−0.086	−0.035	0.062	−0.565	0.574	−0.061
Pragmatic comprehension*Age											−0.053	0.023	−2.294	0.025*	−0.246
*R* ^2^	0.067					0.074					0.134				
*Adjusted R* ^2^	0.055					0.050					0.100				
*F* value	*F* (1,78) = 5.588, *p* = 0.021					*F* (2,77) = 3.080, *p* = 0.052					*F* (3,76) = 3.921, *p* = 0.012				
△*R*^2^	0.067					0.007					0.060				
△*F* value	*F* (1,78) = 5.588, *p* = 0.021					*F* (1,77) = 0.600,*p* = 0.441					*F* (1,76) = 5.262, *p* = 0.025				

Dependable Variable: SPCC *p<0.05 **p<0.01.

Because there was a positive correlation between pragmatic comprehension and SPCC by Model 1 (*p* = 0.021), a moderate effects analysis could be conducted. As Table 7 shows, in Model 3 the interaction between learners’ age and pragmatic comprehension was statistically significant (*t* = −2.294, *p* = 0.025 < 0.05).

Table 8 shows that when learners’ pragmatic comprehension interacts with their SPCC, age plays a moderate role across three levels, of which age at the low and mean level may moderate learners’ SPCC more remarkably (*p* = 0.002; *p* = 0.019).

**TABLE 8:** Simple slope test.

Moderator level	Coefficient of skewness	Std deviation	*t*	*p*	95% CI	
Mean	0.235	0.098	2.399	0.019	0.043	0.426
High(+1SD)	–0.017	0.142	–0.117	0.907	–0.296	0.263
Low(–1SD)	0.486	0.151	3.216	0.002	0.190	0.782

## Discussion

This study examined CSL learners’ pragmatic awareness and pragmatic comprehension, and the possible relation between them and the level of the learners’ WTC and SPCC. The first research question concerned the CSL learners’ degree of pragmatic awareness and pragmatic comprehension. On the one hand, in terms of the pragmatic awareness of CSL learners, our quantitative results showed that more than 60% of the CSL learners performed well in the pragmatic awareness judgment task. The finding indicated that the CSL learners’ pragmatic awareness was rather advanced, which was reasonable considering that most of them had already lived in China for longer than a year. The result is consistent with the findings of previous studies that L2 learners’ pragmatic awareness develops significantly after an academic year spent in the L2 community environment (Bardovi-Harlig and Dörnyei, 1998; Schauer, 2009; Ren, 2015).

The results from the multiple-choice task also revealed high performance by most of the CSL learners in different types of implicature comprehension. In a comparison of subgroups, it was found that the learners interpreted non-conventional implicatures better, indicating that conventional implicatures may be difficult to comprehend. Similarly, Taguchi et al. (2013) also found that conventionally indirect opinions were the most difficult for their learners of Chinese. Thus, it is necessary for CSL instructors to put more emphasis on teaching conventional implicatures in the classroom so that CSL learners’ pragmatic comprehension can be improved. Also, since the target language setting may contribute to the acquisition of conventional implicatures (Inagaki, 2019), CSL learners should take the advantage of their study-abroad opportunity and access more instances of conventional implicatures in daily communication. Due to limited time in the classroom, CSL instructors should also consider the integration of the instructional resources in and outside the classroom into curriculum design (Ren and Han, 2016) and encourage CSL learners to participate in out-of-class activities, as they would benefit CSL learners’ linguistic and pragmatic competence (Gong et al., 2021b; Li et al., 2021). In addition, the study-abroad program would be well-designed to provide more resources and opportunities for CSL learners to communicate with locals so that their pragmatic comprehension and awareness would be well developed (Gong et al., 2018a; Ren, 2019; Li et al., 2021).

The second question asked whether there were correlations between the CSL learners’ pragmatic awareness/pragmatic comprehension and WTC/SPCC. Our statistical analysis did not find a direct correlation between the CSL learners’ pragmatic awareness and WTC. Also, the learners’ pragmatic comprehension did not show a direct correlation with their WTC. This is contrary to the conclusions of Hosseinpur and Nevisi (2017), which indicated a positive relationship between EFL learners’ pragmatic competence and their WTC. A possible interpretation for this finding might be differences in the pragmatic aspects examined and the tasks employed. On the one hand, this study examined CSL learners’ pragmatic awareness and comprehension, while Hosseinpur and Nevisi (2017) examined EFL learners’ pragmatic production. On the other hand, in the present study, the pragmatic awareness judgment task and the pragmatic comprehension test were in the paper-and-pencil version, which may be less affected by learners’ WTC compared to discourse completion task used in Hosseinpur and Nevisi (2017). Therefore, L2 pragmatics research and instruction on learners’ pragmatic production should take full consideration of their WTC. Also, instructors and researchers could develop innovative teaching methods and research methods to encourage CSL learners’ intention to speak, which would benefit their pragmatic competence by developing their WTC. Communicative and intercultural pedagogy can be involved in pragmatics instructions by Chinese language instructors since the teaching intervention would help CSL learners improve their intercultural communicative competence (Moloney, 2013; Gong et al., 2021a). In addition, more pragmatic practices could be provided for CSL learners in and outside the classroom to assist them to improve their pragmatic awareness (Li et al., 2021). Thus, instructional methods should be carefully designed, and instructors’ attitudes are also critical in influencing CSL learners’ WTC.

The findings indicated that the CSL learners’ pragmatic comprehension had a positive correlation with their SPCC. That is, if the CSL learners perceived their communicative competence to be high, they showed better performance in terms of comprehension of implicatures. The construct of communicative competence includes how to utilize pragmatic knowledge to communicate with others (Mao, 2021). Thus, if L2 learners can accurately self-assess their actual communicative competence, they perform well in the comprehension of some implicatures (Jamrus and Razali, 2019). As the findings showed, in this study, SPCC as a predictor of WTC (Burroughs et al., 2003) had a positive correlation with the CSL learners’ comprehension of two subgroups of implicatures, namely conventionally indirect refusals and conventional routines. That is, the learners’ SPCC interacted with their pragmatic comprehension of the conventional implicatures.

Based on previous literature (MacIntyre, 1994; Lahuerta, 2014; Liu, 2016), the present study also conducted an analysis of the moderating effect of variables including gender, age, length of time spent in Chinese-speaking countries, and duration of Chinese learning. However, only the variable of age presented as a moderator of the interaction between pragmatic comprehension and SPCC. This finding echoes previous findings that age has an impact on the development of pragmatic comprehension (Lee, 2010) and on pragmatic production (Liu et al., 2021). In addition, age may also influence language learners’ SPCC. For instance, early starters have been found to self-assess their oral communicative competence more highly than those learning language later (Dewaele, 2009). As L2 pragmatics research predominantly investigate learners at the university level, how age as a moderator promotes the interaction of pragmatic comprehension with SPCC may be an interesting topic for future studies.

## Conclusion and Limitations

This study examined CSL learners’ performance of pragmatic awareness and pragmatic comprehension tasks. Most of the CSL learners attained high scores and performed well in the tasks. The relationships between the learners’ pragmatic awareness/pragmatic comprehension and WTC/SPCC were also examined by correlation analysis. It was found that the CSL learners’ pragmatic comprehension had a positive correlation with their SPCC, but pragmatic awareness and pragmatic comprehension did not correlate with WTC. The study also found age as a moderating factor between the learners’ SPCC and pragmatic comprehension. These findings shed light on our understanding of CSL learners’ individual differences (in terms of their WTC and SPCC) and their pragmatic competence (pragmatic awareness and pragmatic comprehension).

The findings have implications for CSL pragmatics instruction and provide a new perspective on the research of CSL learners’ pragmatic competence. More specifically, this study contributes to research examining the relationship between CSL learners’ pragmatic competence and their WTC. The study suggests that CSL instructors, or L2 instructors in general, should apply the communicative and/or cultural pedagogies in pragmatics teaching and integrate instructional resources and opportunities for CSL learners to develop their SPCC and pragmatic competence. The study also has some limitations. First, we only focused on pragmatic comprehension and awareness. Future studies could also include pragmatic production and compare similarities and differences between productive pragmatic competence and receptive pragmatic competence. Second, a mixed-methods design including interviews or verbal reports (Ren, 2014; Gass and Mackey, 2016) would be helpful to further examine learners’ own understanding of the relationship between pragmatic competence and WTC. Third, instruments could be designed covering several different contexts, taking full consideration of the situated nature of L2 WTC.

## Data Availability Statement

The original contributions presented in the study are included in the article/Supplementary Material, further inquiries can be directed to the corresponding author/s.

## Author Contributions

XL: conceptualization, data analysis, writing, and revision. WR: conceptualization, data collection, data analysis, writing—review, and editing. LL: data collection, data analysis, and revision. All authors contributed to the article and approved the submitted version.

## Conflict of Interest

The authors declare that the research was conducted in the absence of any commercial or financial relationships that could be construed as a potential conflict of interest.

## Publisher’s Note

All claims expressed in this article are solely those of the authors and do not necessarily represent those of their affiliated organizations, or those of the publisher, the editors and the reviewers. Any product that may be evaluated in this article, or claim that may be made by its manufacturer, is not guaranteed or endorsed by the publisher.
