# Supplementary material for: Pragmatic Competence and Willingness to Communicate Among L2 Learners of Chinese
Source: Front Psychol. 2021 Dec 6;12:797419. doi: 10.3389/fpsyg.2021.797419 (PMC8685262; doi:10.3389/fpsyg.2021.797419)
Supplement: Supplementary file 1 [file Data_Sheet_1.docx]

**Appendix I**

**Willingness to Communicate Questionnaire**

Directions: Below are twelve situations in which a person (a stranger, friend, or acquaintance) might choose to communicate or not to communicate. Presume you have completely free choice. Indicate the percentage of time you would choose to communicate in each type of situation. Indicate in the space at the left of the item what percentage of the time you would choose to communicate.

(0 = Never to 100 = Always)

_____1. Present a talk to a group of strangers.

_____2. Talk with an acquaintance in line.

_____3. Talk in a large meeting of friends.

_____4. Talk in a small group of strangers.

_____5. Talk with a friend in line.

_____6. Talk in a large meeting of acquaintances.

_____7. Talk with a stranger in line.

_____8. Present a talk to a group of friends.

_____9. Talk in a small group of acquaintances.

_____10. Talk in a large meeting of strangers.

_____11. Talk in a small group of friends.

_____12. Present a talk to a group of acquaintances.

**Appendix II**

**Self-perceived Communication Competence Questionnaire**

Below are twelve situations in which you might need to communicate. People’s abilities to communicate effectively vary a lot, and sometimes the same person is more competent to communicate in one situation than in another. Please indicate how competent you believe you are to communicate in Chinese in each of the situations described below, using a scale from 0 to 100. Indicate in the space provided at the left of each item your estimate of your competence.

0 = completely incompetent

100 = competent

_____1. Present a talk to a group of strangers.

_____2. Talk with an acquaintance in line.

_____3. Talk in a large meeting of friends.

_____4. Talk in a small group of strangers.

_____5. Talk with a friend in line.

_____6. Talk in a large meeting of acquaintances.

_____7. Talk with a stranger in line.

_____8. Present a talk to a group of friends.

_____9. Talk in a small group of acquaintances.

_____10. Talk in a large meeting of strangers.

_____11. Talk in a small group of friends.

_____12. Present a talk to a group of acquaintances.

**Appendix III**

**Pragmatic Awareness Judgment Task**

There are ten questions in this task. You should judge the appropriateness of the underlined part in each dialogue. If you think it is inappropriate, please rate the inappropriateness from 0 to 6, representing “not bad at all” to “very bad”.

Sample 1 (appropriate expression)

王丽邀请安娜去她家做客，但是安娜去不了。

王丽：安娜，你愿意今天下午过来吗？

安娜：很抱歉，我特别想去，但是我明天下午的历史考试太难了。

划线部分内容表达得体吗？

1. 得体

2. 不得体

如果不得体，您认为程度有多严重？

根本不严重 ○0 ○1 ○2 ○3 ○4 ○5 ○6 非常严重

Maria invites Anna to her house but Anna cannot come.

M: Anna, would you like to come over this afternoon?

A: I’m sorry, I’d really like to come but I have a difficult history test tomorrow.

Is the underlined part in dialogue appropriate?

1. Yes

2. No

If it is inappropriate, how bad do you think it was?

Not bad at all ○0 ○1 ○2 ○3 ○4 ○5 ○6 Very bad

Sample 2 (inappropriate expression)

安娜和王丽约定下午见面，但是安娜很晚才到王丽家。

安娜：你好，王丽。

王丽：你好，安娜。我已经等你半个多小时了。我们不是约定4点见面的吗？

安娜: 我没法早来，反正我们也不着急去哪儿。

划线部分内容表达得体吗？

1. 得体

2. 不得体

如果不得体，您认为程度有多严重？

根本不严重 ○0 ○1 ○2 ○3 ○4 ○5 ○6 非常严重

Anna and Wang Li agreed to meet in the afternoon, but Anna arrived at Wang Li's house very late.

A: Hello, Wang Li.

W: Hello, Anna. I’ve been waiting for over half an hour for you. Weren’t we supposed to meet at 4?

**A: I couldn’t come earlier. And anyway, we don’t have to hurry anywhere.**

Is the underlined part in dialogue appropriate?

1. Yes

2. No

If it is inappropriate, how bad do you think it was?

Not bad at all ○0 ○1 ○2 ○3 ○4 ○5 ○6 Very bad

**Appendix IV**

**Multiple-choice Test for Pragmatic Comprehension**

Direction: There are twelve items in this test. You should choose the best answer based on the conversation.

Sample 1 (non-conventional indirect opinion)

李明和室友杨光正在谈论另一位室友李雷。

李明：杨光，你知道李雷在哪里吗？

**杨光：哦，我刚才好像听见他房间里放音乐了。**

杨光的话是什么意思呢？

A.李雷忘记关掉音乐了。

B.李雷音乐声音太大，影响到了杨光。

C.李雷可能就在他房间里。

D.杨光不知道李雷在哪里。

Li Ming is talking to his housemate Yang Guang about another housemate, Li Lei.

Li Ming: “Do you know where Li Lei is, Yang Guang?”

**Yang Guang: “Well, I heard music from his room earlier.”**

*What does Yang Guang probably mean?*

A. Li Lei forgot to turn the music off.

B. Li Lei’s loud music bothers Li Lei.

C. Li Lei is probably in his room.

D. Yang Guang doesn’t know where Li Lei is.

**Sample 2 (conventional indirect refusal)**

李明和杨光正在一起做一个课程项目，但是他们无法在截止日期前完成。

李明：如果我们不能按时提交的话，你觉得老师会给我们打低分吗？

**杨光：你说呢？**

**杨光的话是什么意思？**

1. 他不知道成绩是否会受到影响。
2. 他认为他们的成绩不会受到影响。
3. 他并不明白李明的问题。
4. 他认为他们的成绩会比较低。

Li Ming and Yang Guang are working on a class project together but they won’t be able to finish it by the deadline.

Li Ming: “Do you think Dr. Gibson is going to lower our grade if we hand it in late?”

**Yang Guang: “What do you think!”**

*What does Yang Guang probably mean?*

A. He did not know whether their grades will be affected.

B. He thinks their grades will not be affected.

C. He did not understand Li Ming’s question.

D. He thinks they will get a lower grade.

**Sample 3 (conventional indirect refusal)**

约翰今天上汉语口语课，他觉得王老师讲课的语速太快，课后他来到老师办公室。

约翰：王老师，您好！您能否在讲课的时候语速慢些？我有点听不懂。

**老师：这是正常语速啊！**

**老师的话是什么意思？**

1. 老师同意下次讲课时放慢语速。
2. 老师认为约翰不适合听这门课程。
3. 老师认为约翰听不懂正常语速的讲话。
4. 老师认为约翰应该适应她的讲课速度。

John was in an Oral Chinese class today and he felt that Mr. Wang was speaking too fast. He came to his teacher’s office after the class.

John: Hello, Mr. Wang! Would you mind speaking more slowly during the lesson? I can’t follow you.

**Teacher: This is the normal speed of speech!**

*What does the teacher probably mean?*

A. The teacher agrees to slow down the pace of the next lecture.

B. The teacher thinks that John is not suitable for the course.

C. The teacher thinks that John cannot understand normal speech.

D. The teacher thinks that John should adapt to the speed of her lectures.
